# IFI204 Restricts *Mannheimia haemolytica* Pneumonia via Eliciting Gasdermin D-Dependent Inflammasome Signaling

**DOI:** 10.3390/microorganisms13112557

**Published:** 2025-11-09

**Authors:** Jia-Qi Li, Yi Zhao, Zhen-Yu Li, Yu-Jing Wu, Xue Chen, Ming-Yue Zhang, Zi-Jian Zhuang, Ao-Bo He, Shu-Xin Zhang, Qian Xu, Ping Sheng, Shui-Xing Yu

**Affiliations:** State Key Laboratory of Reproductive Regulation and Breeding of Grassland Livestock, College of Life Sciences, Inner Mongolia University, Hohhot 010070, China; 15847769721@163.com (J.-Q.L.); 13804752339@163.com (Y.Z.); 15534230622@163.com (Z.-Y.L.); 18547710072@163.com (Y.-J.W.); 15069819017@163.com (X.C.); zhangmy0622@163.com (M.-Y.Z.); 18147104563@163.com (Z.-J.Z.); heaobchuannong@163.com (A.-B.H.); zsx1230205@163.com (S.-X.Z.); xq15174941516@163.com (Q.X.); 15548448539@163.com (P.S.)

**Keywords:** interferon-inducible protein 204, gasdermin D, inflammasome, *Mannheimia haemolytica*, pneumonia

## Abstract

Host innate immunity is crucial for orchestrating a protective response against dangerous pathogens. Herein, we demonstrate that interferon-inducible protein (IFI204), a DNA sensor, is implicated in protection against pulmonary pathogenic *Mannheimia haemolytica* (*M. haemolytica*) infection by driving inflammasome signaling activation. *Ifi204^−/−^* mice are more susceptible to pathogenic *M. haemolytica* infection compared with their wild-type (WT) counterparts, with decreased survival rates, extensive lung architecture destruction, exacerbated inflammatory cells infiltration, and more bacterial colonization. In vivo and in vitro findings elucidate that *Ifi204* deficiency leads to a defect in inflammasome signaling activation, and exogenous recombinant IL-18 is sufficient to rescue the susceptibility of *Ifi204^−/−^* mice. Inflammasome signaling downstream of IFI204 facilitates early bacterial killing and clearance. Mechanistically, IFI204 promotes gasdermin D (GSDMD)-dependent inflammasome activation, and GSDMD is required for IFI204-mediated host defense. Notably, IFI204 detects pathogenic *M. haemolytica*-derived genomic DNA for the inflammasome signaling response. Thus, these data highlight the requirement of IFI204 in host defense response to *M. haemolytica* infection, and reveal that IFI204 may be a potential therapeutic target for pathogen control.

## 1. Introduction

*Mannheimia haemolytica* (*M. haemolytica,* previously known as *Pasteurella haemolytica*) is an opportunistic pathogen that causes fibrinonecrotic pneumonia in cattle and is the primary bacterial agent in bovine respiratory disease syndrome (BRD) [1,2,3]. Approximately 30% of BRD-related cattle deaths are attributed to *M. haemolytica*, causing over $1 billion in annual economic losses in North America [4]. Additionally, *M. haemolytica* can induce mastitis, pneumonia, acute gastroenteritis, and septicemia in goats and sheep, posing significant global threats to the livestock industry [5,6]. Although traditional antibiotic therapies are widely used, concerns are growing over multidrug-resistant strains [7,8]. While vaccines offer a desirable prophylactic alternative, their efficacy is limited by variable or ineffective cross-immunoprotection across serotypes [9,10,11]. Therefore, it is essential to develop novel strategies for the control of pathogenic *M. haemolytica* infections.

The innate immune system is the first line of host defense against invading pathogens. Non-self and endogenous danger signals from microbial infection or tissue damage are sensed through interactions with pattern recognition receptors (PRRs) called pathogen-associated molecular patterns (PAMPs) and damage-associated molecular patterns (DAMPs) [12]. It is generally accepted that PRRs in mammals include the following categories: Toll-like receptors (TLRs), RIG-like receptors (RLRs), NOD-like receptors (NLRs), C-type lectin receptors (CLRs), and a range of intracellular DNA sensors [13]. Therefore, it is not surprising that clarification of the host innate sensing mechanisms paves the way in the development of more effective prevention and control strategies to counter *M. haemolytica* epidemic.

The interferon-inducible protein 204 (IFI204), the murine homolog of human IFI16, functions as an intracellular DNA receptor that can sense invading viruses and bacteria [14,15,16]. Recent research has demonstrated that active IFI204 promotes type I IFN signaling and extracellular traps formation, in response to pathogenic infection [17,18,19]. In addition, although previous work has reported that IFI204 does not recognize intracellular Lipopolysaccharide (LPS) and is dispensable for inflammasome activation [20], there is evidence showing that IFI204 recruits apoptosis-associated speck-like protein containing a caspase recruitment domain (ASC) to form an inflammasome, resulting in the production of pro-inflammatory cytokines [21,22]. Indeed, the biological implications of IFI204 have been discussed, although there is little evidence regarding the effect of IFI204 in response to pulmonary *M. haemolytica* infection.

In our study, we reveal the protective role of IFI204 in host defenses against *M. haemolytica* infection. Compared with their wild-type counterparts, *Ifi204*^−/−^ (*Ifi204*-deficient) mice display increased bacterial loads, reduced survival rates, and more severe organ damage. Importantly, inflammasome signaling downstream of IFI204 facilitates bacterial killing and clearance. In addition, IFI204 detects pathogenic *M. haemolytica*-derived genomic DNA for the inflammasome signaling response. Thus, our results indicate that IFI204 is critical for host defense against *M. haemolytica* infection, which provides a reference for the subsequent development of an efficient bovine respiratory disease syndrome vaccine.

## 2. Materials and Methods

### 2.1. Mice and Cells

*Ifi204*-deficient (*Ifi204*^−/−^) mice and *Gsdmd*-deficient (*Gsdmd*^−/−^) mice were generously given by Prof. Yong-jun Yang (Jilin University, Changchun, China). All the mice were housed in SPF-grade, independently ventilated cages (IVCs) located at the Laboratory Animal Center of Inner Mongolia University (Hohhot, China). All experimental procedures involving animals reported in this study were granted approval by the Animal Welfare and Research Ethics Committee of Inner Mongolia University ([2022] 072, 1 January 2022). Matured bone-marrow-derived macrophages (BMDMs) were acquired from the femurs of 6–8-week-old mice, and cultured in the RPMI-1640 medium (Gibco, #31800-022, Waltham, MA, USA) containing 10% fetal bovine serum (FBS, Gibco, #A31608-02), 25% L929 cell-conditioned medium, and 100 U/mL penicillin/streptomycin (P/S, Gibco, #15140-122) to differentiate as previously reported [23].

### 2.2. Phylogenetic Assay

Bacterial genomic DNA was extracted via the *EasyPure*^®^ Bacteria Genomic DNA Kit (TransGen Biotech, Beijing, China, #EE161-11), and 16S rRNA genes were amplificated and subjected to phylogenetic analysis via the maximum likelihood (ML) method using Mega 11 software [24].

### 2.3. In Vivo Infection

*M. haemolytica* strain MH-1, a clinical isolate of pathogenic *M. haemolytica* (specific 16S rDNA gene sequence data deposited in NCBI GenBank: PQ268926) was cultured at 37 °C in brain–heart infusion (BHI, Qingdao Hi-Tech Industrial Park Haibo Biotechnology Co., Ltd., Qingdao, China, #HB8297-5) medium. To induce pneumonia, six-to-eight-weeks-old sex-matched mice were intranasally infected with 5 × 10^9^ colony-forming units (CFU) of log-phase pathogenic *M. haemolytica* strain MH-1 (day 0), and the lungs, blood, and bronchoalveolar lavage fluid (BALF) were aseptically collected for the quantification of bacterial loads at 24 h post-infection (hpi, day 1). To administer the exogenous recombinant IL-18 (rIL-18, Novoprotein, Summit, NJ, USA, #CK06), rIL-18 (1.0 µg per mouse) was injected intraperitoneally on day 1 and day 0. For survival experiments, mice were intranasally infected with 8 × 10^9^ CFU of log-phase pathogenic *M. haemolytica* strain MH-1, and their mortality was monitored over 72 h.

### 2.4. Histopathology and Immunostaining

For the histology, aseptically excised lungs were fixed in 4% paraformaldehyde (PFA, Macklin, Shanghai, China, #P804536), and the lung sections were stained with hematoxylin and eosin (H&E, Solarbio, Beijing, China, #SL7050-500). For the immunohistochemistry, the lung sections were stained with anti-Gr-1 (Abcam, Cambridge, UK, #ab196436), anti-F4/80 (BioLegend, San Diego, CA, USA, #123119), and anti-IFI204 (Abcam, #ab307201) antibodies.

### 2.5. Inflammasome Assays

Matured BMDMs were stimulated with 500 ng/mL LPS (Invitrogen, Carlsbad, CA, USA, #tlrl-3pelps) for 4 h prior to their challenge with pathogenic *M. haemolytica* strain MH-1 (MOI = 50, 5 h) or their transfection with bacterial genomic DNA (50 µg/mL, 8 h). The BMDMs supernatants and extracts were used for immunoblotting, ELISA, and LDH activities analysis.

### 2.6. Protein Extraction and Immunoblotting

Infected lungs or BMDMs extracts were precipitated by methanol/chloroform for analysis using immunoblot, as previously described [25]. Subsequently, they were incubated with the indicated anti-Caspase-1 (Adipogen, Liestal, Switzerland, #AG-20B-0042-C100), anti-GSDMD (Santa Cruz, Dallas, TX, USA, #sc-393656), anti-IFI204 (Abcam, #ab307201), and anti-GAPDH (Roteintech, Rosemont, IL, USA, #60004-1-Ig) antibodies.

### 2.7. Cytokine and LDH Activities Detection

Aseptically excised lungs homogenates, BALF, and BMDMs supernatants were measured via an ELISA assay, following the manufacturer’s protocols, using a IL-1β ELISA kit (R&D, Minneapolis, MN, USA, #MLB00C) and TNF-α ELISA kit (R&D, #MTA00B). For LDH activities detection, the BMDMs supernatants were determined using the LDH Cytotoxicity Assay kit^®^ (Beyotime Biotechnology, Shanghai, China, #C0017) in accordance with the manufacturer’s instructions.

### 2.8. Immunofluorescence

ASC speckles and IFI204 staining were detected using an indirect immunofluorescence method [26]. Stimulated BMDMs were incubated with anti-ASC (Adipogen, #A29151803f), Alexa Fluor 594-conjugated anti-mouse IgG (Invitrogen, #ab150116), anti-IFI204 (Abcam, #ab307201), and Alexa Fluor 488-conjugated anti-rabbit IgG (Invitrogen, #ab150077) antibodies. DAPI (Solarbio, #C0065) was used to stain nuclei.

### 2.9. Bacterial Killing Analysis

To determine the bacterial killing capacity of BMDMs, LPS-primed BMDMs were incubated with rIL-18 (1000 pg/mL) or PBS for 1 h, before being infected with *M. haemolytica* strain MH-1 (MOI = 20, 6 h). The cells’ supernatants were collected and plated on BHI agar plates to enumerate the bacteria after their overnight culture.

### 2.10. Statistical Analysis

The data were expressed as mean ± standard deviation (SD) and performed using Prism software (GraphPad Software, version 8.0.2, La Jolla, CA, USA). A one-way ANOVA (Dunnett’s *t*-test), unpaired Student’s *t*-test or log-rank test were used for group comparisons. *p*-values that were less than 0.05 were regarded as statistically significant (* *p* < 0.05 and ** *p* < 0.01).

## 3. Results

### 3.1. IFI204 Is Critical for Host Defense Against Pulmonary Pathogenic M. haemolytica Infection

**Figure 1:**
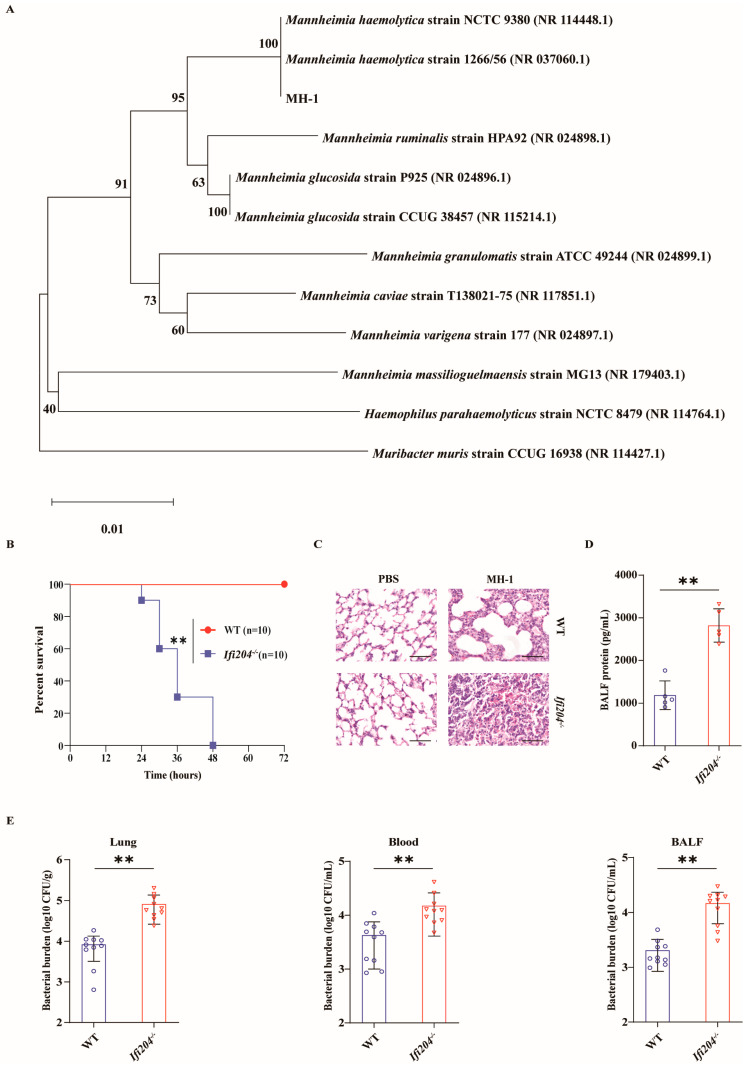
**IFI204 is sufficient to protect against *M. haemolytica* pulmonary infection.** (**A**) Neighbor-joining phylogenetic tree showing the relative position of the isolated *M. haemolytica* strain MH-1 based on the 16S rDNA sequences. Age- and sex-matched WT and *Ifi204^−/−^* mice (n = 10) were intranasally challenged with log-phase pathogenic *M. haemolytica* strain MH-1. (**B**) Survival rate (8 × 10^9^ CFU, *p* < 0.0001). (**C**) Representative H&E staining of the lung tissue structures (5 × 10^9^ CFU, at 24 hpi, magnification, ×400). (**D**) Total protein in BALF was determined (5 × 10^9^ CFU, at 24 hpi, *p* = 0.0001). (**E**) Bacterial loads in the lungs (*p* = 0.0006), blood (*p* = 0.0079) and BALF (*p* = 0.0002) were assessed (5 × 10^9^ CFU, at 24 hpi). Graphs are means ± standard deviation (SD) from data pooled from ten (**B**,**E**) and five (**D**) biological replicates. Statistical significance is considered as ** *p* < 0.01.

To explore the biological role of IFI204 in pulmonary host protection against pathogenic *M. haemolytica* infection in vivo, WT and *Ifi204^−/−^* mice were intranasally infected with 8 × 10^9^ CFU of log-phase pathogenic *M. haemolytica* strain MH-1, a clinical isolate of pathogenic *M. haemolytica* (16S rDNA sequencing analysis, Figure 1A), and the survival rate of animals was monitored for 72 h. Clearly, *Ifi204^−/−^* mice displayed significantly higher mortality rates compared with their WT counterparts (Figure 1B). To further characterize the phenotype of *Ifi204^−/−^* mice in response to pathogenic *M. haemolytica*, WT and *Ifi204^−/−^* mice were intranasally challenged with 5 × 10^9^ CFU of log-phase pathogenic *M. haemolytica* for 24 h post-infection (hpi). In accordance with their decreased survival rate, *Ifi204^−/−^* mice also tended to acquire extensive pulmonary damage, destroyed pulmonary architecture, and disrupted barrier function (Figure 1C,D). Based on the above results, we speculated that the increased mortality and exacerbated pathology in *Ifi204^−/−^* mice during pathogenic *M. haemolytica* infection might link to increased bacterial load levels. As expected, *Ifi204^−/−^* mice harbored significantly elevated loads of pathogenic *M. haemolytica* in the lungs, blood, and bronchoalveolar lavage fluid (BALF) (Figure 1E). Therefore, these results reveal that IFI204 contributes to host defense against pathogenic *M. haemolytica* pneumonia.

### 3.2. IFI204-Elicited Inflammasome Signaling Confers Protection Against Pulmonary Pathogenic M. haemolytica Infection

**Figure 2:**
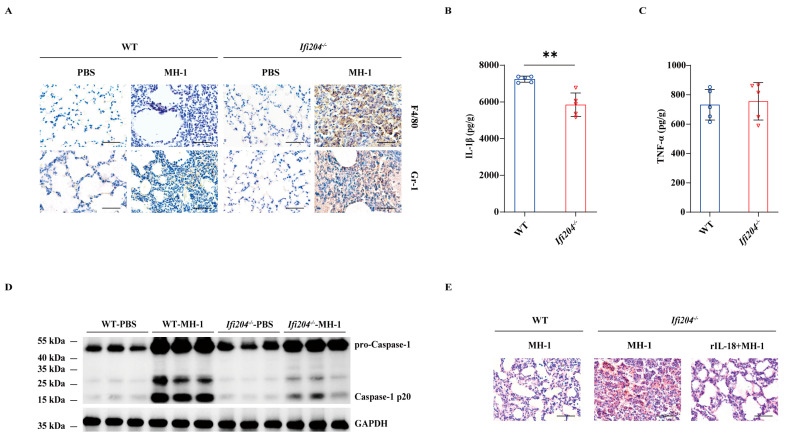
**Involvement of the inflammasome signaling response in IFI204-mediated host defense.** Age- and sex-matched WT and *Ifi204^−/−^* mice were intranasally stimulated with 5 × 10^9^ CFU log-phase pathogenic *M. haemolytica* strain MH-1 for 24 h. (**A**) Representative infected lung sections. Infiltrated inflammatory cells were stained brown (IHC, magnification, ×400). (**B**,**C**) Pro-inflammatory cytokines IL-1β (*p* = 0.0014) and TNF-α (*p* = 0.7533) production in the homogenate supernatants of infected lung tissue were determined by ELISA. (**D**) Caspase-1 activation was examined in the homogenate lysate of infected lung tissue by immunoblotting. For one group of *Ifi204^−/−^* mice, rIL-18 (1 μg/mouse) was intraperitoneally injected prior to intranasally challenged with 5 × 10^9^ CFU log-phase pathogenic *M. haemolytica* strain MH-1 for 24 h. (**E**) Representative H&E staining of the lung tissue structures (magnification, ×400). Graphs are means ± standard deviation (SD) from data pooled from five (**B**,**C**) biological replicates. Statistical significance is considered as ** *p* < 0.01.

To assess the potential immunological role of IFI204 in host protection, we next evaluated the lungs’ inflammatory responses. Although neutrophils and macrophages accumulation dramatically elevated in the lung tissue of infected *Ifi204^−/−^* mice relative to the level observed in those of the infected WT controls (Figure 2A), the inflammatory cytokine IL-1β release was markedly suppressed in *Ifi204^−/−^* mice (Figure 2B), while TNF-α production was little affected (Figure 2C). Importantly, inflammasome-dependent Caspase-1 cleavage was also dramatically attenuated in the lung tissue of infected *Ifi204^−/−^* mice compared with their infected WT counterparts (Figure 2D and Figure S1), indicating that *Ifi204* deficiency impaired inflammasome signaling activation during the pulmonary pathogenic *M. haemolytica* challenge. Subsequently, the study further determined whether aberrant inflammasome signaling accounts for the aggravated susceptibility of *Ifi204^−/−^* mice to pathogenic *M. haemolytica* infection. Exhilaratingly, infected *Ifi204^−/−^* mice displayed obviously improved pathological damage in their lung tissue when prophylactically administrated with exogenous recombinant IL-18 (rIL-18) (Figure 2E). Thus, these data showed that inflammasome signaling may be involved in IFI204-mediated host defense against pathogenic *M. haemolytica* infection.

### 3.3. IFI204 Promotes GSDMD-Dependent Inflammasome Activation

**Figure 3:**
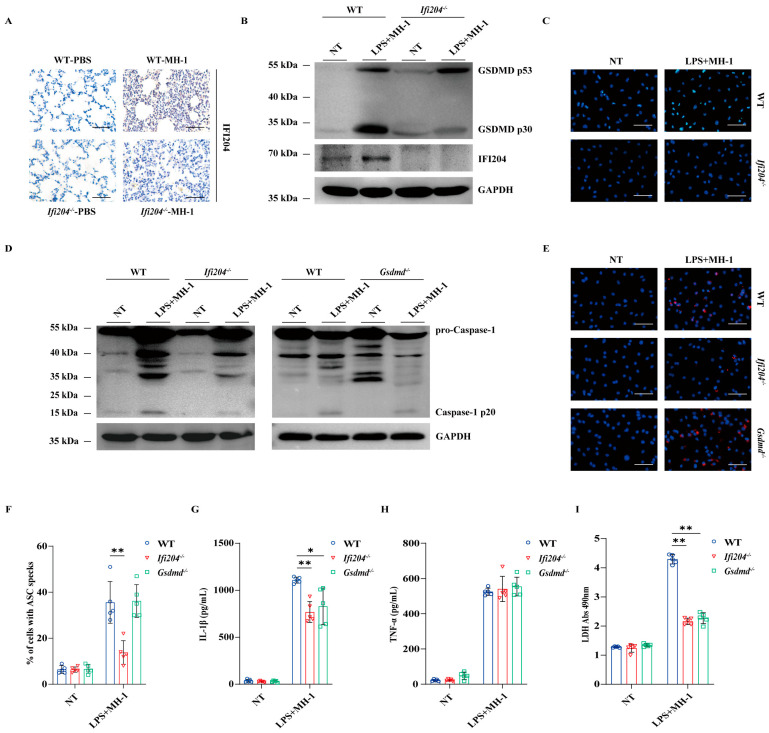
**IFI204 drives inflammasome signaling activation in an GSDMD-dependent manner.** Age- and sex-matched WT and *Ifi204^−/−^* mice were intranasally challenged with 5 × 10^9^ CFU log-phase pathogenic *M. haemolytica* strain MH-1 for 24 h. (**A**) Representative infected lung sections. IFI204 expression was stained brown (IHC, magnification, ×400). LPS-pretreated WT, *Ifi204^−/−^* and *Gsdmd^−/−^* BMDMs were exposed to log-phase pathogenic *M. haemolytica* (MOI = 50, 5 h). (**B**,**D**) Cleaved GSDMD and IFI204 in cell lysates (Lys.), as well as cleaved Caspase-1 in culture supernatants (Sup.) were detected by immunoblotting. (**C**,**E**,**F**) IFI204 expression, ASC speckles formation (*p* = 0.0016, *p* = 0.9842) and quantification were measured by immunofluorescence (magnification, ×400). (**G**,**H**) IL-1β (*p* = 0.00017, *p* = 0.0014) and TNF-α (*p* = 0.7583, *p* = 0.2690) secretion in the BMDMs supernatants were indicated by ELISA. (**I**) LDH released in the BMDMs supernatants were determined (*p* < 0.0001, *p* < 0.0001). Graphs are means ± standard deviation (SD) from data pooled from five (**F**–**I**) biological replicates. Statistical significance is considered as * *p* < 0.05, ** *p* < 0.01.

To dissect the IFI204-driven host defense signaling mechanisms, the positive expression location of IFI204 was detected in lung sections. Immunohistochemical (IHC) staining revealed that elevated expression of IFI204 was observed in the infiltrated inflammatory cells of the infected tissues (Figure 3A). Correspondingly, the IFI204 protein level was also prominently up-regulated by the pathogenic *M. haemolytica* challenge in bone marrow-derived macrophages (BMDMs) (Figure 3B,C and Figure S2). Subsequently, we asked if *Ifi204* deficiency impairs inflammasome signaling in macrophages. Consistent with the in vivo findings, inflammasome signaling was significantly attenuated in *Ifi204^−/−^* BMDMs vs. in WT BMDMs, as shown by the weakened Caspase-1 cleavage, impaired ASC speckles formation, reduced LDH activity, and mature IL-1β secretion (Figure 3D–G,I and Figure S3), while TNF-α secretion was not significantly affected (Figure 3H), suggesting that *Ifi204* deficiency leads to a defect in inflammasome signaling activation in response to pathogenic *M. haemolytica* infection. Especially, gasdermin D (GSDMD), a critical executor of inflammasome activation, was also evidently suppressed in *Ifi204^−/−^* BMDMs (Figure 3B). Importantly, IL-1β release and LDH activity were remarkably inhibited in *Gsdmd^−/−^* BMDMs following pathogenic *M. haemolytica* challenge (Figure 3G,I), despite Caspase-1 activation, ASC speckles formation and TNF-α production being little affected (Figure 3D–F,H). These results demonstrated that IFI204 activates inflammasome signaling in a GSDMD-dependent manner.

### 3.4. GSDMD Is Required for IFI204-Mediated Host Defense

**Figure 4:**
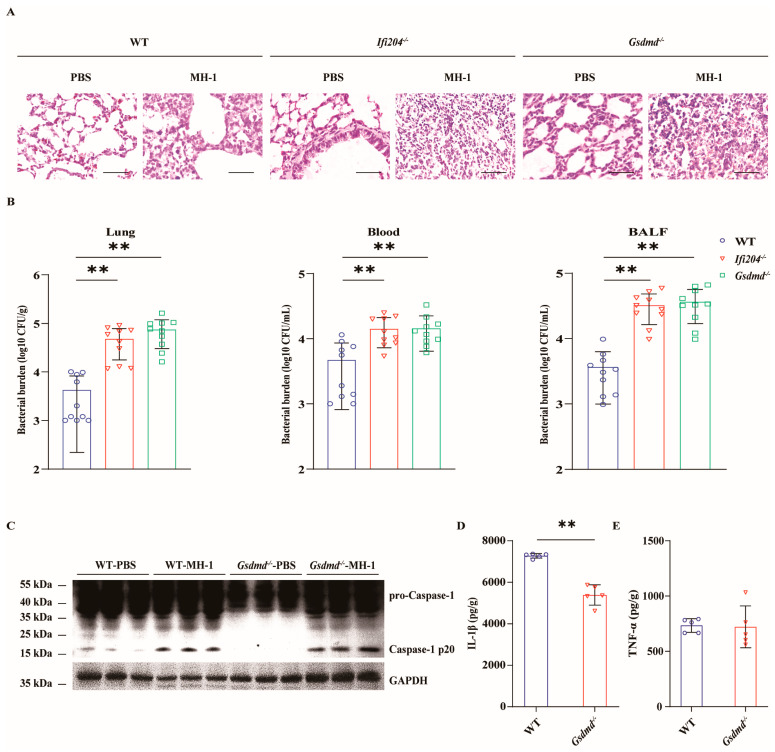
***Gsdmd* deficiency impairs host protection against *M. haemolytica* pulmonary infection.** Age- and sex-matched WT, *Ifi204^−/−^* and *Gsdmd^−/−^* mice (n = 10) were intranasally challenged with 5 × 10^9^ CFU log-phase pathogenic *M. haemolytica* strain MH-1 for 24 h. (**A**) Representative H&E staining of the lung tissue structures (magnification, ×400). (**B**) Bacterial loads in the lungs (*p* = 0.0003, *p* < 0.0001), blood (*p* = 0.0014, *p* = 0.0028), and BALF (*p* < 0.0001, *p* < 0.0001) were enumerated. (**C**) Caspase-1 activation was examined in the homogenate lysate of infected lung tissue via immunoblotting. (**D**,**E**) Pro-inflammatory cytokines IL-1β (*p* < 0.0001) and TNF-α (*p* = 0.8934) production in the homogenate supernatants of infected lung tissue were determined via ELISA. Graphs are means ± standard deviation (SD) from data pooled from ten (**B**) and five (**D**,**E**) biological replicates. Statistical significance is considered as ** *p* < 0.01.

To further characterize whether inflammasome-associated GSDMD mediates the protective effect of IFI204, the susceptibility of *Gsdmd^−/−^* mice in response to pathogenic *M. haemolytica* infection was evaluated in vivo. In line with *Ifi204^−/−^* mice, *Gsdmd^−/−^* mice were more susceptible to pathogenic *M. haemolytica* infection compared with WT mice. The date show that *Gsdmd^−/−^* mice exhibited severe histological damage in their lungs (Figure 4A). Specifically, *Gsdmd^−/−^* mice harbored higher bacterial loads in their lungs, blood, and BALF (Figure 4B), indicating that *Gsdmd* deficiency results in the defect of pathogen clearance in host defense responses. In addition, *Gsdmd* deficiency also impaired the inflammasome signaling activation following pathogenic *M. haemolytica* infection (Figure 4C–E and Figure S4), which was consistent with in vitro findings. Altogether, these data showed that inflammasome-activated GSDMD is beneficial to host protection against pulmonary pathogenic *M. haemolytica* infection.

### 3.5. IFI204-Driven Inflammasome Signaling Facilitates Pathogen Control

**Figure 5:**
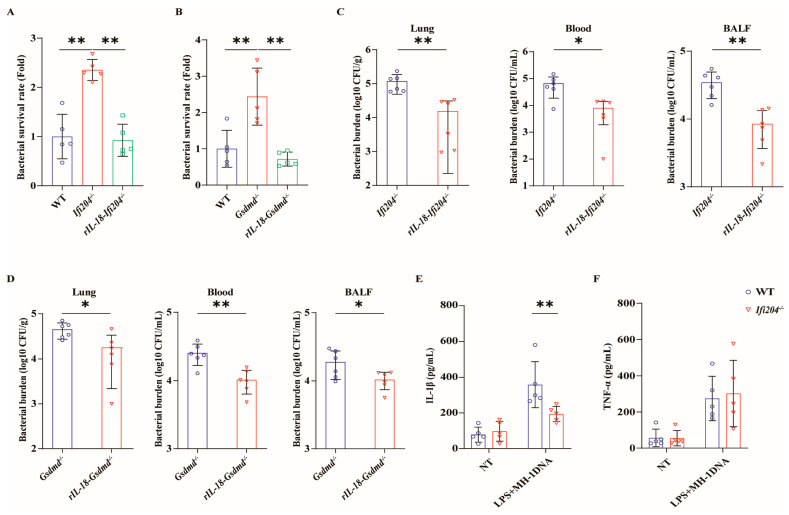
**Inflammasome signaling downstream of IFI204 restricts bacterial invasion.** LPS-pretreated WT, *Ifi204^−/−^*, and *Gsdmd^−/−^* BMDMs were primed with rIL-18 (1 ng/mL, 1 h) or PBS before being stimulated with *M. haemolytica* (MOI = 20, 6 h). (**A**,**B**) The bacterial survival was assessed (*p* = 0.0003, *p* < 0.0001). Age- and sex-matched *Ifi204^−/−^* and *Gsdmd^−/−^* mice were intraperitoneally injected with rIL-18 (1 μg/mouse) or PBS prior to being intranasally challenged with 5 × 10^9^ CFU log-phase pathogenic *M. haemolytica* strain MH-1 for 24 h (*p* = 0.0089, *p* = 0.0014). (**C**,**D**) Bacterial loads in the lungs (*p* = 0.0053, *p* = 0.0181), blood (*p* = 0.0413, *p* = 0.0031), and BALF (*p* = 0.0019, *p* = 0.042) were determined. (**E**,**F**) LPS-pretreated WT and *Ifi204^−/−^* BMDMs were exposed to *M. haemolytica*-derived genomic DNA (50 µg/mL, 8 h). IL-1β (*p* = 0.0072) and TNF-α (*p* = 0.9167) secretion in the BMDMs supernatants were indicated via ELISA. Graphs are means ± standard deviation (SD) from data pooled from five (**A**,**B**,**E**,**F**) and six (**C**,**D**) biological replicates. Statistical significance is considered as * *p* < 0.05, ** *p* < 0.01.

Based on the above, *Ifi204^−/−^* mice and *Gsdmd^−/−^* mice harbored increased tissue bacterial loads, suggesting that there is a defect in pathogen control in the absence of *Ifi204* and *Gsdmd* during pulmonary pathogenic *M. haemolytica* infection. We set out to characterize the bacterial killing capacity of *Ifi204^−/−^* BMDMs and *Gsdmd^−/−^* BMDMs in vitro. Similarly, the results revealed that a higher number of recovered viable bacteria were observed in *Ifi204^−/−^* BMDMs and *Gsdmd^−/−^* BMDMs compared with WT BMDMs following pathogenic *M. haemolytica* challenge (Figure 5A,B), indicating that *Ifi204* and *Gsdmd* deficiency also impair the bacterial killing capacity of macrophages. Since inflammasome signaling activation is required for host innate immune defenses against pathogenic invasion, we further evaluated the role of IFI204-mediated inflammasome signaling on bacterial growth and proliferation. To our delight, exogenous rIL-18 could strongly decrease the bacterial loads in the lungs, blood, and BALF of *Ifi204^−/−^* mice and *Gsdmd^−/−^* mice, and could rescue the defect in bacterial killing in *Ifi204^−/−^* BMDMs and *Gsdmd^−/−^* BMDMs, respectively (Figure 5A–D). These results show that inflammasome signaling downstream of active IFI204 accelerates bacterial killing and clearance. Then, to further illustrate how pathogenic *M. haemolytica* drives IFI204 signaling, *Ifi204^−/−^* BMDMs and WT BMDMs were incubated with pathogenic *M. haemolytica*-derived genomic DNA. Notably, *Ifi204* deficiency attenuated pathogenic *M. haemolytica*-derived genomic DNA-triggered IL-1β production, while TNF-α release was not significantly affected (Figure 5E,F). Collectively, our findings reported that the DNA sensor IFI204 promotes pathogen control via eliciting GSDMD-dependent inflammasome signaling in response to pulmonary pathogenic *M. haemolytica* infection.

## 4. Discussion

*M. haemolytica* pneumonia is one of the most economically important infectious diseases of ruminants, with a wide prevalence throughout the continents [27]. What is worse, with the emergence of multidrug-resistant *M. haemolytica* strains [28,29], the development of new prophylaxis and treatment strategies has become more urgent. Correspondingly, a *M. haemolytica* wild-type isolate MH-1 has previously been isolated and confirmed. Detailed understanding of *M. haemolytica*–host interactions is central in controlling this infection. In the current study, we find that IFI204 promotes bacterial killing and clearance by driving GSDMD-dependent inflammasome signaling, and we indicate that therapeutic interventions targeting IFI204 may show a clinical benefit in combating *M. haemolytica* infections.

Indeed, *Ifi16*, the homolog of murine *Ifi204* expression, is reported in various ruminants [30,31]. To investigate the possible involvement of IFI204 in the host response to *M. haemolytica* infection in vivo, using a *M. haemolytica* pneumonia model, this study observed that *Ifi204^−/−^* mice exhibited increased bacterial loads, decreased survival, and severe destruction of lung architecture, as expected, indicating that IFI204 contributes to host protection against *M. haemolytica* pulmonary infection. In line with this, IFI204/IFI16 has also been implicated in response to intracellular and extracellular bacterial infections including *Francisella novicida* [14], *Listeria monocytogenes* [32], *Mycobacterium bovis* [15], and *Staphylococcus aureus* [18,19]. Altogether, this finding extends and highlights the potential immunological and biological role of IFI204 in pathogens infections.

IFI204/IFI16 is an intracellular innate immune receptor that functions as a DNA sensor, recognizing pathogen-derived double-stranded DNA to activate inflammasome signaling [33,34] and interacting with STING to trigger type I IFN signaling upon cytosolic DNA detection [35,36]. To elucidate IFI204-mediated host defense mechanisms, we assessed its role in inflammatory responses. Notably, *M. haemolytica* pulmonary infection induced a significantly higher release of IL-1β and Caspase-1 cleavage in WT mice compared to *Ifi204^−/−^* mice, indicating IFI204’s involvement in *M. haemolytica*-induced inflammasome activation—a pathway responsible for IL-1β and IL-18 processing [37,38]. Further investigation into inflammasome-dependent IL-1 family members revealed their critical role in resistance. Crucially, exogenous rIL-18 administration strongly protected infected *Ifi204^−/−^* mice, evidenced by reduced bacterial burden and attenuated lung damage. Collectively, these results show that inflammasome signaling downstream of IFI204 confers resistance to *M. haemolytica* infection.

Given the prominent polymorphonuclear neutrophils (PMNs) infiltration and predominant IFI204 localization within recruited inflammatory cells following *M. haemolytica* infection, we employed BMDMs to investigate the molecular mechanisms of IFI204-mediated inflammasome activation. Evidence shows IFI16/IFI204-mediated inflammasome activation via bacterial infections, like *Campylobacter concisus* [39,40], and via viral pathogens, such as KSHV and HIV [34,41,42,43]. p204 (IFI204) recruits ASC, via its N-terminal PYD domain, upon cytosolic dsDNA sensing, to form an inflammasome [21,44]. However, IFI204-mediated anti-infective signaling in *M. haemolytica* infection remains poorly characterized. We found that *M. haemolytica* induces IFI204 expression in BMDMs, and *Ifi204* deficiency impairs inflammasome signaling, as evidenced by reduced Caspase-1 cleavage, ASC speckle formation, mature IL-1β production, and LDH activity. GSDMD, an executor of cytokine release and pyroptosis [45], was inhibited in the absence of *Ifi204* after the *M. haemolytica* challenge. Hence, our in vivo and in vitro findings demonstrate that GSDMD is involved in M. haemolytica-initiated IFI204-related inflammasome signaling.

Appropriate inflammasome activation is crucial for host defense against bacterial, viral, fungal, and protozoan pathogens [40,46,47,48], promoting immune responses that restrict invasion by pathogens such as *Salmonella*, *Bacillus anthracis*, influenza, *Candida albicans, Clostridium tyzzeri*, and *Francisella tularensis* [49,50,51,52,53,54]. Inflammasome-activated gasdermin D (N-terminal cleavage product, GSDMD-NT) directly kills both Gram-negative (e.g., *E. coli*) and Gram-positive (e.g., *S. aureus*, *L. monocytogenes*) bacteria [55]. Similarly to *Ifi204^−/−^* mice, we then utilized *Gsdmd^−/−^* mice to further demonstrate that GSDMD facilitates host survival and bacterial control during *M. haemolytica* pulmonary infection. Exogenous rIL-18 rescues the susceptibility of *Gsdmd^−/−^* mice to *M. haemolytica* infection in vivo and the defect in bacteria clearance of *Ifi204^−/−^* and *Gsdmd^−/−^* BMDMs in vitro, highlighting the importance of IFI204-modulated inflammasome signaling in response to *M. haemolytica* invasion. Given that IFI204/IFI16 functions as an innate immune sensor of pathogens and host DNA in the cytoplasm and nucleus [56,57,58,59], we investigated if *M. haemolytica* genomic DNA triggers IFI204 signaling. Notably, *Ifi204* deficiency significantly suppressed bacterial genomic DNA-induced mature IL-1β release, whereas TNF-α level was unchanged, indicating that *M. haemolytica* genomic DNA potentially promotes IFI204-modulated inflammasome signaling. While the mechanisms underlying IFI204 activation by *M. haemolytica* require further investigation, our findings enhance understandings of IFI204’s immunological role in host defense.

In conclusion, we demonstrate here that IFI204/IFI16 plays a nonredundant role in restricting *M. haemolytica* pneumonia, through activating the GSDMD-dependent inflammasome signaling. We also show that bacterial genomic DNA may be a critical factor that induces IFI204/IFI16-elicited inflammasome signaling activation. Therefore, it is of future interest to evaluate whether pharmacological target of IFI204/IFI16 signaling confers protection in pathogenic infectious diseases.

## Data Availability

The original contributions presented in this study are included in the article/Supplementary Material. Further inquiries can be directed to the corresponding author.

## Supplementary Materials

The following supporting information can be downloaded at: https://www.mdpi.com/article/10.3390/microorganisms13112557/s1, Figure S1: Quatification of Caspase-1 p20 determined by densitometry of protein bands from three experiments.The GAPDH served as a loading control (*p* < 0.0001).Graphs are means ± standard deviation (SD), Statistical significance is considered as ** *p* < 0.01. Figure S2: Quatification of Caspase-1 p20 and IFI204 determined by densitometry of protein bands from three experiments.The GAPDH served as a loading control (*p* < 0.0001).Graphs are means ± standard deviation(SD), Statistical significance is considered as ** *p* < 0.01. Figure S3: Quatification of Caspase-1 p20 determined by densitometry of protein bands from three experiments. The GAPDH served as a loading control (*p* < 0.0001, *p* = 0.1205). Graphs are means ± standard deviation (SD), Statistical significance is considered as ** *p* < 0.01. Figure S4: Quatification of Caspase-1 p20 determined by densitometry of protein bands from three experiments. The GAPDH served as a loading control (*p* = 0.5604). Graphs are means ± standard deviation (SD), Statistical significance is considered as ** *p* < 0.01.
