# Supplementary figures and images for: IFI204 Restricts Mannheimia haemolytica Pneumonia via Eliciting Gasdermin D-Dependent Inflammasome Signaling
Source: Microorganisms. 2025 Nov 9;13(11):2557. doi: 10.3390/microorganisms13112557 (PMC12654538; doi:10.3390/microorganisms13112557)

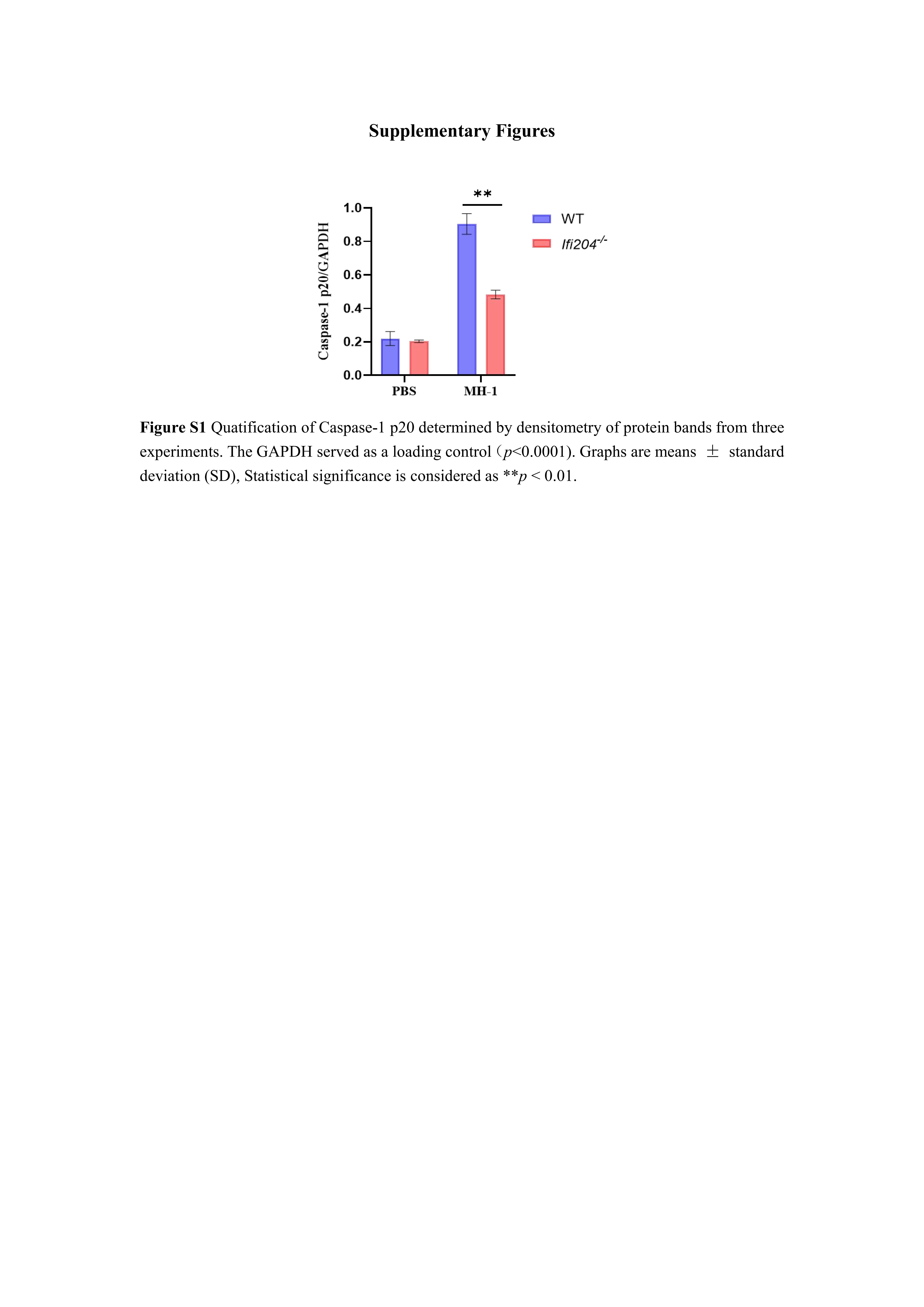

Supplement: Supplementary file 1 [file microorganisms-13-02557-s001.zip › Figure S1.jpg]

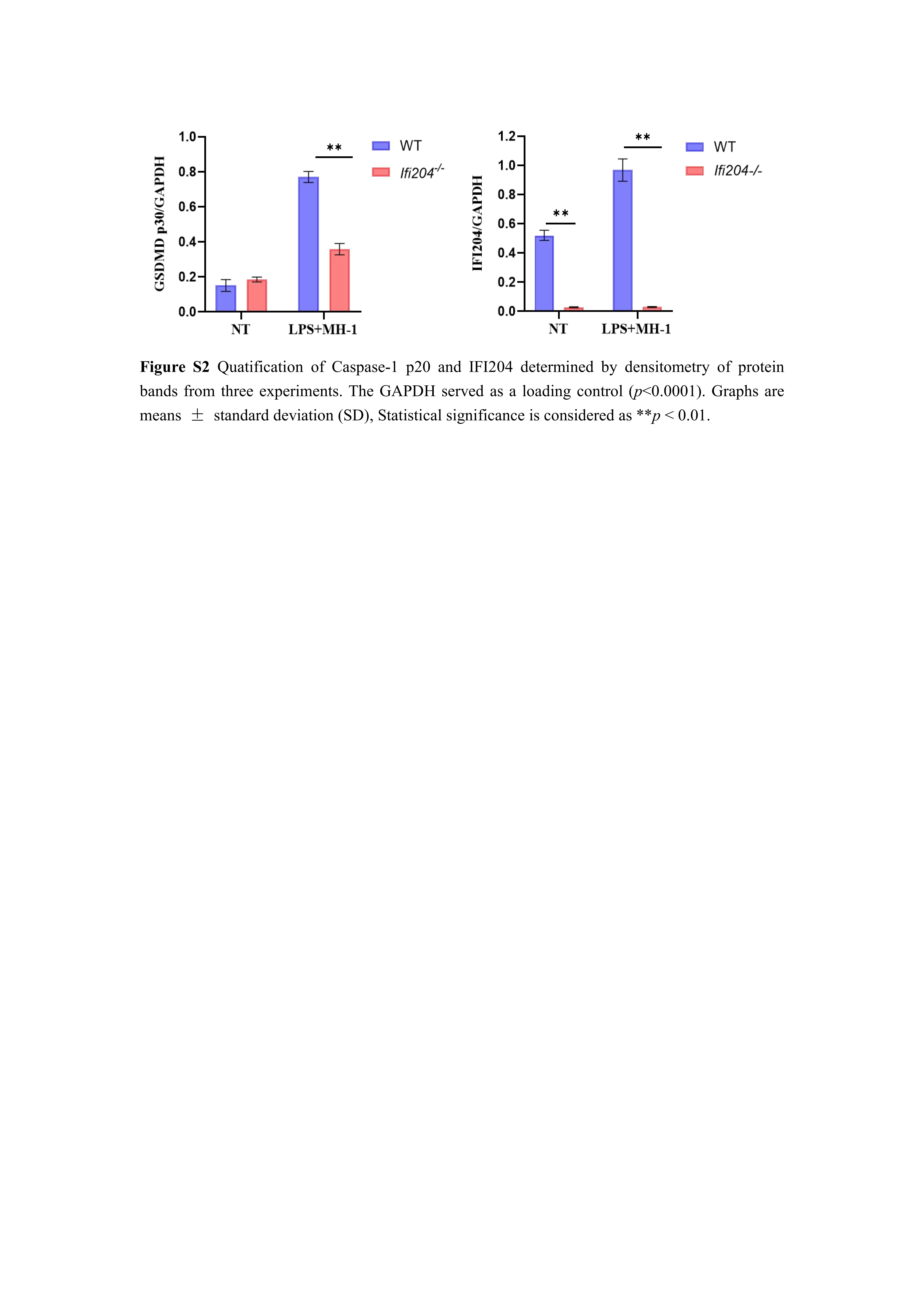

Supplement: Supplementary file 1 [file microorganisms-13-02557-s001.zip › Figure S2.jpg]

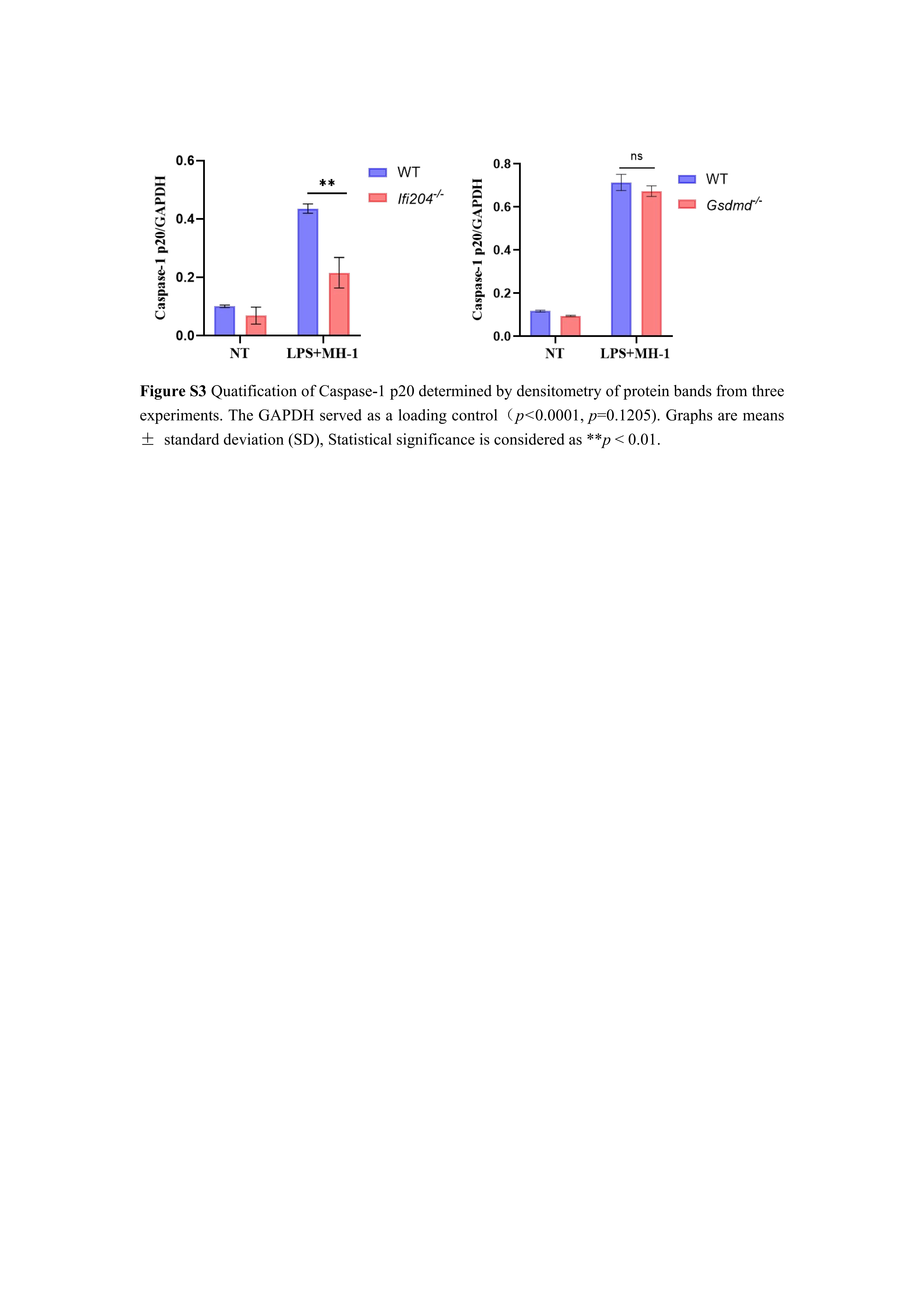

Supplement: Supplementary file 1 [file microorganisms-13-02557-s001.zip › Figure S3.jpg]

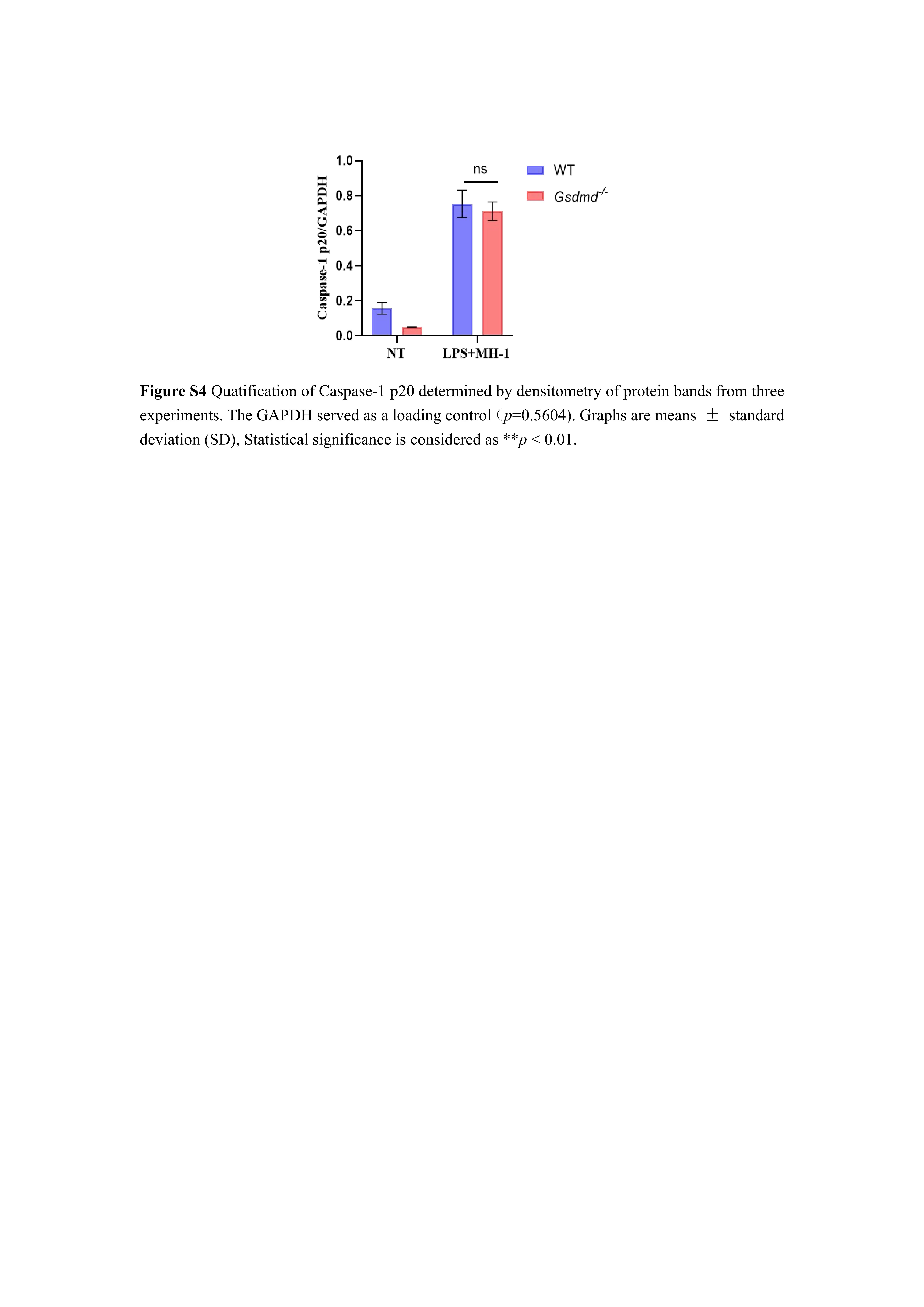

Supplement: Supplementary file 1 [file microorganisms-13-02557-s001.zip › Figure S4.jpg]
